# Small Non-Coding RNA Profiling Identifies miR-181a-5p as a Mediator of Estrogen Receptor Beta-Induced Inhibition of Cholesterol Biosynthesis in Triple-Negative Breast Cancer

**DOI:** 10.3390/cells9040874

**Published:** 2020-04-03

**Authors:** Elena Alexandrova, Jessica Lamberti, Pasquale Saggese, Giovanni Pecoraro, Domenico Memoli, Valeria Mirici Cappa, Maria Ravo, Roberta Iorio, Roberta Tarallo, Francesca Rizzo, Francesca Collina, Monica Cantile, Maurizio Di Bonito, Gerardo Botti, Giovanni Nassa, Alessandro Weisz, Giorgio Giurato

**Affiliations:** 1 Laboratory of Molecular Medicine and Genomics, Department of Medicine, Surgery and Dentistry ‘Scuola Medica Salernitana’, University of Salerno, 84081 Baronissi, Italy; 2 Genomix4Life Srl, 84081 Baronissi, Italy; 3 Division of Pulmonary and Critical Care Medicine, David Geffen School of Medicine, University of California, Los Angeles, CA 90095, USA; 4 Pathology Unit, Istituto Nazionale Tumori-IRCCS-Fondazione G. Pascale, 80131 Naples, Italy; 5 Scientific Direction, Istituto Nazionale Tumori-IRCCS-Fondazione G. Pascale, 80131 Naples, Italy

**Keywords:** triple-negative breast cancer, estrogen receptor beta, small non-coding RNAs, microRNA, cholesterol biosynthesis

## Abstract

Triple-negative breast cancer (TNBC) is a highly heterogeneous disease, representing the most aggressive breast cancer (BC) subtype with limited treatment options due to a lack of estrogen receptor alpha (ERα), progesterone receptor (PR), and Erb-B2 receptor tyrosine kinase 2 (HER2/neu) expression. Estrogen receptor beta (ERβ) is present in a fraction of TNBC patients, where its expression correlates with improved patient outcomes, supported by the fact that it exerts oncosuppressive effects in TNBC cell models in vitro. ERβ is involved in microRNA-mediated regulation of gene expression in hormone-responsive BC cells and could mediate its actions through small noncoding RNAs (sncRNAs) in TNBCs also. To verify this possibility, smallRNA sequencing was performed on three ERβ-expressing cell lines from different TNBC molecular subtypes. Several sncRNAs resulted modulated by ERβ, with a subset being regulated in a tumor subtype-independent manner. Interestingly, sncRNA profiling of 12 ERβ+and 32 ERβ− primary TNBC biopsies identified 7 microRNAs, 1 PIWI-interacting RNA (piRNA), and 1 transfer RNA (tRNA) differentially expressed in ERβ+ compared to ERβ− tumors and cell lines. Among them, miR-181a-5p was found to be overexpressed in ERβ+ tumors and predicted target key components of the cholesterol biosynthesis pathway previously found to be inhibited by ERβ in TNBC cells.

## 1. Introduction

Triple-negative breast cancer (TNBC) is the most aggressive breast cancer (BC) subtype, responsible for 25% of breast cancer-related deaths [1]. Its phenotype is characterized by high heterogeneity, early-onset, rapid growth, poor survival, and high recurrence and metastases rates [2]. TNBC tissues do not express estrogen receptor alpha (ERα), progesterone receptor (PR), and epidermal growth factor receptor 2 (HER2/neu) [3]; this is why these tumors are immune to hormonal and HER2-targeting therapies commonly used for breast cancer treatment. Currently, the most frequently used therapy for TNBC treatment is cytotoxic chemotherapy, aimed at killing TNBC cancer cells [4], which however often leads to the development of drug resistance [5]. For this reason, new therapeutic approaches against these aggressive tumors are much sought after.

Estrogen signaling plays a key role in BC carcinogenesis and it is mediated by two receptors belonging to the nuclear receptor superfamily: ERα and ERβ, encoded by the *ESR1* and *ESR2* genes, respectively [6,7], that play opposite roles in hormone-responsive breast cancer progression. Indeed, both in vivo and in vitro studies demonstrated that ERα expression increases cellular proliferation and positively controls epithelial–mesenchymal transition (EMT) whereas ERβ exerts anti-proliferative effects and inhibits EMT [8]. It is also known that ERβ expression is frequently lost in mammalian epithelial cells during malignant transformation, even though it is expressed at higher levels than ERα in both human and mouse normal mammary glands [9]. However, the role of ERβ in BC is still unclear as, in addition to full-length ERβ, C-terminally truncated receptor isoforms are expressed in breast cancer tissues, where they exert pro-proliferative effects [10]. Another factor hindering ERβ research is the poor specificity of antibodies raised against this protein, especially the ones that recognize the C-terminal part of the receptor, generally spliced to form truncated receptor isoforms [11,12]. In any case, full-length receptor expression was reported in a small fraction (15–20%) of TNBC patients, where its presence was correlated with better survival [13] and response to tamoxifen therapy [13], suggesting its possible use as both a prognostic marker and therapeutic target [14]. In accordance with this data, in our previous study [15] we demonstrated the oncosuppressive role of the full-length ERβ in three TNBC cell lines belonging to different TNBC subtypes. 

Small non-coding RNAs (sncRNAs) are RNA molecules of 200 nucleotides or less in length that include the following short RNA subclasses: microRNAs (miRNAs), PIWI-interacting RNAs (piRNAs), transfer RNAs (tRNAs), small nuclear RNAs (snRNAs), and small nucleolar RNAs (snoRNAs) [16]. Among them, miRNAs are involved in post-transcriptional regulation of gene expression by gene silencing through inhibition of gene translation or mRNA degradation [17] and represent the most studied group of sncRNAs. miRNAs are known regulators of the following fundamental biological processes: cell proliferation, differentiation, migration, invasion, and apoptosis [17,18]. Moreover, they play an important role in carcinogenesis, as confirmed by miRNA deregulation in all cancer types [19] and may therefore be useful as diagnostic and prognostic biomarkers of these diseases [20]. Finally, the fact that miRNAs are secreted from cancerous tissues and are found in the blood stream of patients as free molecules or enclosed inside extracellular vesicles makes liquid biopsy miRNA profiling an appealing non-invasive diagnostic tool in BC [21]. ERβ involvement in miRNA-mediated gene regulation in hormone-responsive BC cells has been previously reported [22,23,24], suggesting that this nuclear receptor may exert similar effects in TNBC also, a possibility worth exploring given the importance of sncRNAs in BC cell biology.

To verify this hypothesis and investigate the role of ERβ in TNBC, we performed sncRNA sequencing on three previously engineered receptor-expressing cell lines and on 12 ERβ+ and 32 ERβ− TNBC tissue samples where receptor status was assessed by immunohistochemistry [15]. A group of ERβ-regulated sncRNAs was identified both in vitro and in vivo, several of which showed subtype-specific deregulation, while others were independent from the tumor subtype. Interestingly, two miRNAs—miR-181a-5p and miR-92a-3p—showed the same response to the receptor in all cell lines and tissues tested. Among them, miR-181a-5p was characterized by high expression and upregulation in TNBC tissues and cell lines and was found to target key components of the cholesterol biosynthesis pathway, previously shown to be inhibited by ERβ in TNBC cells by ERβ-mediated recruitment of transcriptional repressor complexes to regulatory elements of cholesterol biosynthesis genes [15]. Taken together, these findings suggest a dual role of ERβ in epigenetic regulation of gene expression in TNBC: at the transcriptional level via chromatin binding and recruitment of multiple chromatin-modifying complexes to the genome, and post-transcriptionally through sncRNA-mediated regulation of gene expression.

## 2. Materials and Methods

### 2.1. Ethics Approval and Consent to Participate

The study protocol received approval by the Ethics Committee of the Istituto Nazionale Tumori ‘Fondazione Giovanni Pascale’ (protocol n.er CEI/393/15) before the beginning of the study, in accordance with The code of Ethics of the Declaration of Helsinki, and informed consent was obtained from all patients involved.

### 2.2. TNBC Cell Line Maintenance and ERβ Clone Generation

Triple-negative breast cancer cell lines HCC1806 (CRL-2335), MDA-MB-468 (HTB-132), and Hs 578T (HTB-126) were purchased from the American Type Culture Collection (ATCC, Manassas, VA, USA) and maintained in culture as previously described [15]. Lentiviral particles containing viral RNA genome encoding for Tet-On Advanced and Inducible 3xFlag-ERβ were produced using Lenti-X Tet-On Advanced Inducible Expression System components (Takara-Clontech Europe, Göteborg, Sweden) according to the manufacturer’s instructions. The aforementioned TNBC cell lines were then transduced with Tet-On Advanced-encoding lentiviral particles, and separate Tet-On Advanced clones were grown in the presence of selective antibiotic G418 (Gibco-Thermo Fisher Scientific, Waltham, MA, USA). One Tet-On Advanced clone for each cell line was chosen for further transduction using 3xFlag-ERβ-encoding lentiviral particles followed by single clone isolation in the presence of puromycin (Sigma-Aldrich, St Louis, MO, USA). Separate clones were tested for ERβ expression after 24-h induction of transgene expression by 2 µg/mL doxycycline (Sigma-Aldrich) as previously described [15]. For each cell line, one ERβ clone expressing the receptor at a maximum level was chosen for further experiments.

### 2.3. The Cancer Genome Atlas (TCGA) Data Analysis

Data from the TCGA Breast Cancer dataset [25] for mRNA expression were downloaded from the cBioPortal data source. A subgroup of 122 samples, negative for the expression of ERα, PR, and HER2/neu, was filtered out from the dataset and further considered as TNBC samples. Survival analyses were performed using the survminer R package.

### 2.4. Immunohistochemistry Assay

An immunohistochemistry assay of 44 TNBC samples was performed as previously described [15]. Briefly, the most representative inclusion of TNBC samples was used for tissue microarray construction. Formalin-fixed paraffin-embedded (FFPE) sections were deparaffinized and immunohistochemically stained using mouse monoclonal anti-ERβ antibody (PPZ0506, Thermo Fisher Scientific). Both qualitative and quantitative parameters were evaluated for the determination of ERβ positivity. The intensity of the nuclear staining (“mild”, “moderate”, or “intense”) was taken into account as the qualitative criteria, whereas the percentage of positive tumor cells was considered for the quantitative criteria.

### 2.5. Protein Extraction and Western Blotting

Total protein extraction was performed using RIPA buffer (50 mM Tris-HCl pH 7.6, 150 mM NaCl, 0.1% SDS, 0.5% C_24_H_39_NaO_4_, 1% NP-40, 2 mM EDTA, and 50 mM NaF). Briefly, cells were grown in the presence of 0.035, 1, and 2 μg/mL doxycycline for HCC1806, Hs 578T, and MDA-MB-468 ERβ-expressing clones, respectively, for 9 days, and were then harvested, washed with PBS-EDTA (0.5 mM EDTA), and lysed for 15 min on ice. After that, samples were centrifuged for 30 min at 13,000 rpm at 4 °C and supernatants were collected, quantified using Bradford protein assay, and resuspended in Laemmli buffer (4% SDS, 20% glycerol, 10% 2-mercaptoethanol, 0.004% bromphenol blue and 0.125 M Tris HCl, pH 6.8). Standard protocols were used for SDS-PAGE and Western blotting with the following antibodies: anti-β-actin (A1978, Sigma-Aldrich) and anti-ERβ PPZ0506 (MA5-24807, Thermo Fisher Scientific).

### 2.6. RNA Isolation and Quality Controls

Total RNA was extracted using TRIzol Reagent (Sigma-Aldrich) from cells maintained for 9 days in doxycycline-containing medium (ERβ+) as previously described [15]. As a control, RNAs from the same cell lines grown in the absence of doxycycline (ERβ−) were isolated. Total RNA extraction from TNBC FFPE tissues was instead performed using the miRNeasy FFPE kit (Qiagen, Hilden, Germany). Both extraction methods were performed according to the manufacturers’ protocols. Then, 5 µg of each cell-line derived RNA sample were treated with TURBO DNase (Thermo Fisher Scientific) in the presence of Murine RNAse inhibitor (NEB, Ipswich, MA, USA). RNA concentration and purity were evaluated using NanoDrop™ 2000/2000c (Thermo Fisher Scientific), whereas sample integrity was analyzed by Agilent 2100 Bioanalyzer (Agilent, Santa Clara, CA, USA) using the RNA 6000 Nano kit or by TapeStation 2200 (Agilent, Santa Clara, CA, USA) using RNA ScreenTape Assay.

### 2.7. sncRNA Sequencing and Data Analysis

Here, 1 µg of each FFPE- or cell line-derived sample was used for sequencing library preparation using TruSeq Small RNA kit (Illumina Inc., San Diego, CA, USA) as previously described [26]. Each library was sequenced on HiSeq 2500 (Illumina Inc., San Diego, CA, USA) at a concentration of 10 pM for 50 cycles plus seven additional cycles for index sequencing in the single-read mode (1 × 50 base pairs). The sequences were filtered based on the minimum length of 15 bp. Reads that had a read count less than three were excluded. SncRNA sequencing data analysis was performed integrating iSmaRT (integrative Small RNA Tool-kit) [27] and SPAR (Small RNA-seq Portal for Analysis of sequencing expeRiments) [28], allowing for the identification of the following small non-coding RNA classes: microRNA (miRBase v21, genome assembly GRCh37/hg19 [29]), piRNA (piRNABank [30]), and tRNA (UCSC Genome Browser [31]), present in the sample. Moreover, the analysis was expanded to other sncRNA types, including the ones present in such databases as Rfam [32], RefSeq [33], and DashR v2.0 [34]. To identify differentially expressed sncRNAs in ERβ+ vs ERβ− cell lines or tissues, the DESeq2 version 1.26.0 algorithm was used [35]. SncRNAs whose expression changed in ERβ+ vs ERβ−, were statistically significant (*p* ≤ 0.05), and were characterized by |Fold-Change| (|FC|) ≥ 1.3, were considered as differentially expressed between the two conditions.

miRNA target prediction was performed using miRWalk v.3.0 [36], which calculates a score for each putative miRNA–mRNA interaction. As highly predicted miRNA targets, we considered only mRNAs characterized by a prediction score equal to 1 and identified by at least two out of four algorithms present in miRWalk database. For cell lines, differentially expressed mRNAs with |FC| ≥ 1.5 were considered as putative miRNA targets, taking into account both experimentally validated and highly predicted interactions, as done in our previous paper [26]. For the prediction of miRNA targets in TNBC tissues, publicly available RNA-seq data from the TCGA Breast Cancer dataset [25] were downloaded and all predicted miRNA-targeted RNAs were identified and taken into account, excluding transcripts that were not expressed in the tissues. The lists of mRNA targets were submitted to Ingenuity Pathway Software (IPA, Ingenuity System) and analysis of deregulated canonical pathways and functions was carried out.

Data integration, heatmaps demonstrating differentially expressed sncRNAs, and functional enrichment plots were prepared using the Multi Experiment Viewer software v4.9 [37] and R/Bioconductor packages, respectively [38]. Raw sncRNA sequencing data are deposited in the EBIArrayExpress database with accession number E-MTAB-8807.

## 3. Results

### 3.1. Characterization of Small Non-Coding RNA Expression Profile of Triple-Negative Breast Cancer Cell Lines

**Figure 1:**
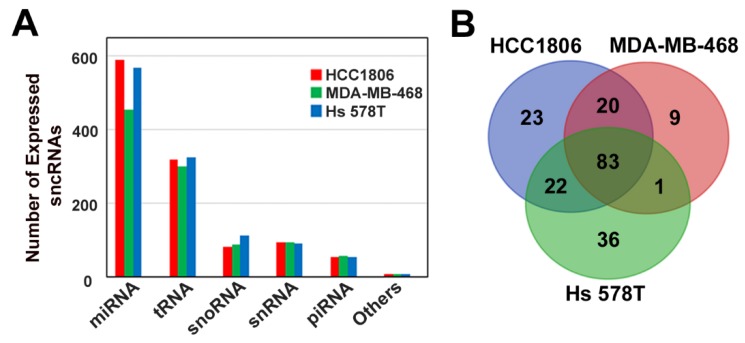
Characterization of small noncoding RNA (sncRNA) expression profiles in triple-negative breast cancer (TNBC) cell lines. (**A**) Bar plot showing the number of expressed microRNAs (miRNAs), transfer RNA (tRNAs), small nucleolar RNAs (snoRNAs), small nuclear RNAs (snRNAs), PIWI-interacting RNA (piRNAs), and other small noncoding RNAs (sncRNAs) in the indicated cell lines. Only sncRNAs whose expression level exceeded three normalized reads are reported. (**B**) Venn diagram showing the number of common and specific miRNAs, highly expressed (expression level above the third quartile of the normalized read-count) in TNBC cell lines.

To investigate the sncRNA expression profile of TNBC cell lines, we performed small non-coding RNA profiling of the MDA-MB-468, HCC1806, and Hs 578T cell lines, which belong to the basal-like 1, basal-like 2, and claudin-low TNBC subtypes, respectively. In line with expectations, the analysis of sncRNA distribution between different subclasses revealed that in all three TNBC cell subtypes, the highest number of small RNA molecules was represented by miRNAs, followed by tRNAs, snoRNAs, snRNAs, and piRNAs (Figure 1A). A comparison of the number of expressed sncRNA molecules between the tested cell lines demonstrated that they were similar for all sncRNA subtypes, except for miRNAs, whose total number was lower in MDA-MB-468 (453 miRNAs) when compared to the other two cellular models (591 and 569 for HCC1806 and Hs 578T cells, respectively). Focalizing the attention on the highly expressed miRNAs (expression level above the third quartile in all cell lines), we observed that the majority (83 molecules) were common for all cell lines. For miRNAs the expression values (normalized reads) were between 703 and 458,000, with an average of 22,500; for tRNAs the values ranged from 321 to 19,355, with an average of 977; for snoRNAs the values ranged from 241.3 to 41,130, with an average of 3029; for snRNAs the values ranged from 578 to 30,351, with an average of 4323; and for piRNAs the values ranged from 448 to 3478, with an average of 1534. Analysis of highly expressed miRNA distribution among cell lines revealed that a group of 20 such RNAs was present in either basal-like cells or basal-like 2 and claudin low cell lines, whereas 9, 23, and 36 miRNAs showed a TNBC histotype-specific expression and were found in MDA-MB-468, HCC1806, and Hs 578T cells, respectively (Figure 1B, Supplementary Figure S1). A comparison of the top expressed tRNAs, snoRNAs, snRNAs, and piRNAs revealed similar result, with a share of the highly expressed molecules (46, 13, 14, and 9 tRNAs, snoRNAs, snRNAs, and piRNAs, respectively) common for all cell lines (Supplementary Figure S2). We further analyzed whether any of the highly expressed miRNAs were previously associated with TNBC and found that seven out of the 83 miRNAs, common for all three cell lines (miR-181b-5p, miR-221-3p, miR-27a-3p, miR-21-3p, miR-20a-5p, miR-103a-3p and miR-25-3p), have been previously associated with the TNBC phenotype [39,40,41,42,43,44,45,46,47] Another three molecules, miR-210-3p, miR-155-5p and miR-125b-5p, highly expressed in HCC1806 and Hs 578T cells, are known as hallmarks of TNBC [40,41,48,49], whereas miR-342-3p, highly expressed exclusively in basal-like TNBC cell lines, has been previously described as an important regulator of molecular mechanisms of this breast cancer subtype [50]. Interestingly, miRNAs miR-101-3p, miR-17-5p, miR-93-5p, miR-340-5p, and miR-31-5p, known mainly for their tumor suppressor properties, were found among the top expressed miRNAs in our dataset [51,52,53,54,55,56,57,58]. Finally, three miRNAs whose role is contradictory according to the literature (miR-181a-5p, miR-182-5p, and miR-26a-5p) were found to be highly expressed in all three cell lines analyzed [39,59,60,61,62,63,64,65,66,67,68]. Altogether, the analysis of sncRNA expression profile in wild-type MDA-MB-468, HCC1806, and Hs 578T TNBC cell lines confirm that the data generated here represent a valuable data source that may be used for further studies.

### 3.2. Association of ERβ mRNA Expression and Overall Survival of Breast Cancer Patients

**Figure 2:**
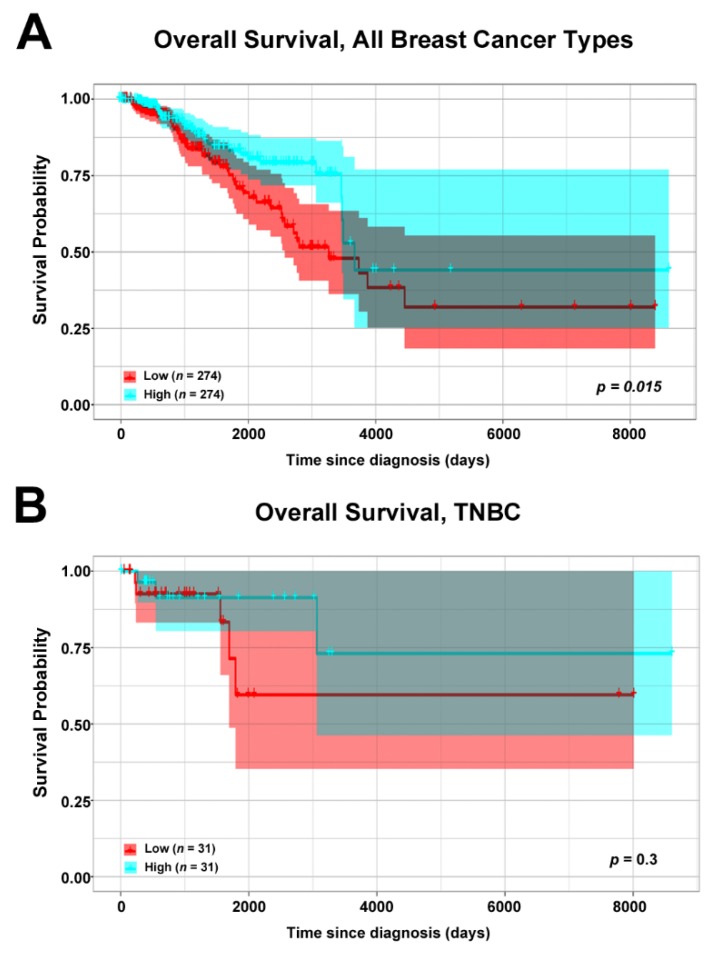
Estrogen receptor beta (ERβ) mRNA expression and survival of breast cancer patients from The Cancer Genome Atlas (TCGA) cohort [25]. Kaplan–Meier curves of overall survival for breast cancer (BC) (**A**) and TNBC (**B**) patients with respect to ERβ mRNA expression level (low expression: values below the first quartile, high expression: values above the third quartile). The highlighted area indicates confidence interval.

In our previous study, using a validated anti-ERβ antibody for immunohistochemistry experiment [12], we demonstrated that ERβ is expressed in a fraction of TNBC tissues [15]. As the role of this receptor in breast cancer is still debated, in order to shed light on its functions in this cancer type we performed a correlation analysis of the ERβ mRNA expression level with the overall survival of breast cancer patients. To this aim, we mined breast cancer RNA-Seq data publicly available in The Cancer Genome Atlas (TCGA) [25], and stratified all samples in two groups: one with low ERβ mRNA expression level (below first quartile), and the other with high ERβ mRNA expression (above third quartile). In accordance with previously published data [15], correlation analysis of all breast cancer patients’ overall survival (OS) and receptor expression demonstrated that patients expressing high levels of ERβ mRNA had a better prognosis (Figure 2A), indicating the possibility that ERβ may exert similar effects also in TNBC. To verify this hypothesis, we performed the correlation analysis using the same patient cohort, from which positive ERα, PR, and HER2/neu samples were excluded. In this case, patients characterized by high ERβ mRNA expression showed a tendency for better survival (Figure 2B) which was not statistically significant, probably due to the almost ten times lower number of samples used for the analysis (31 sample vs 274 used for the first analysis), suggesting anti-proliferative ERβ effects also in TNBC tissues.

### 3.3. ERβ Expression Induces a Profound Effect on Small Non-Coding RNA Profile in Triple-Negative Breast Cancer Cells

**Figure 3:**
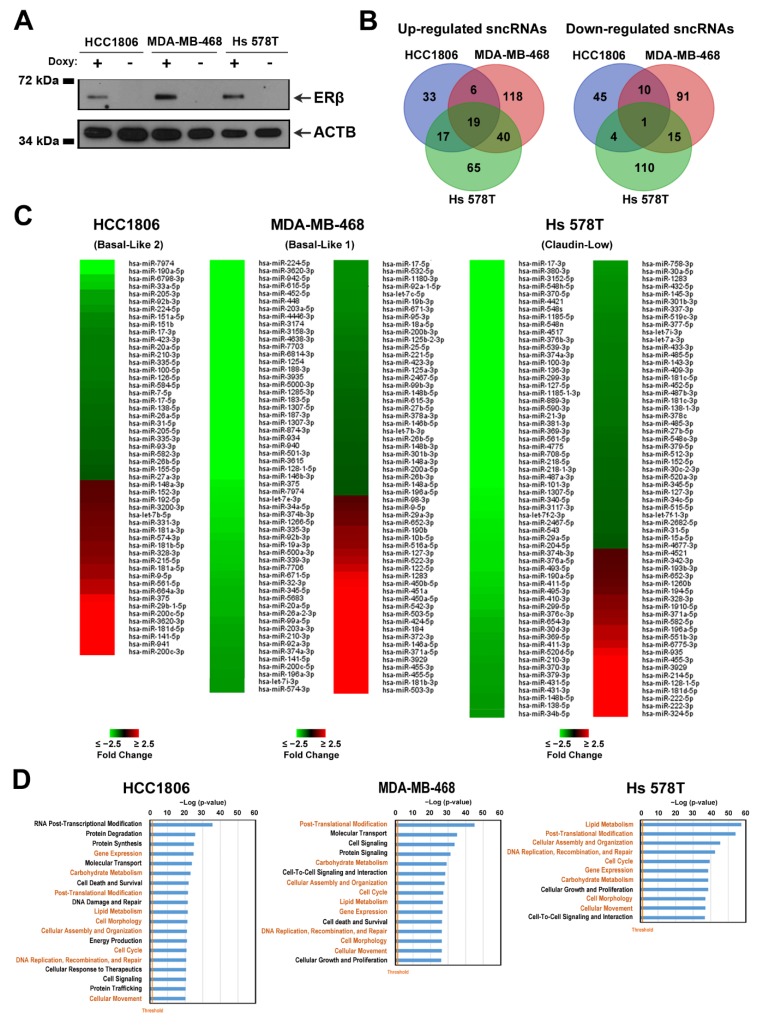
Effect of ERβ on sncRNA expression profiles and miRNA-regulated functions in TNBC. (**A**) Western blot analysis of ERβ expression in the HCC1806, MDA-MB-468, and Hs 578T cell lines upon induction of exogenous receptor expression by doxycycline (Doxy+) or in its absence (Doxy−). (**B**) Venn diagram of specific and commonly up-regulated (left panel) or down-regulated (right panel) sncRNAs in ERβ-expressing TNBC cells (|Fold-Change|(|FC|) ≥ 1.5, *p* < 0.05). Only sncRNAs characterized by the same behavior (up- or down-regulated) were included. (**C**) Heatmap showing ERβ-regulated miRNAs in the indicated cell lines (|FC| ≥ 1.5, *p* < 0.05) (**D**) Ingenuity Pathway Software (IPA) functional annotation analysis performed on ERβ-modulated genes predicted to be targets of differentially expressed miRNAs (|FC| ≥ 1.5, *p* < 0.05) in the corresponding cell lines. Commonly influenced functions in the three cell lines are indicated in orange. The vertical orange line indicates the *p* threshold (*p* < 0.05).

In our previous study, by using HCC1806, MDA-MB-468, and Hs 578T cell lines we generated cell culture models that express inducible ERβ [15]. Analysis of the receptor effects on main cellular processes revealed that its presence inhibits growth, migration, and colony formation, accompanied by reduced cell cycle kinetics due to accumulation of cells in G1 phase [15]. Additional studies, performed on hormone-responsive breast cancer cells, demonstrated that ERβ presence regulates miRNA expression [69] and miRNA loading to RNA-induced silencing complex (RISC) through interaction with RISC components [24], suggesting that ERβ may have a similar role also in TNBC. To elucidate this, we assessed ERβ-specific modulation of the sncRNA profile in three receptor-expressing cell lines. To this aim, total RNA was isolated from clones that were grown for 9 days in the presence or absence of doxycycline, with a concentration sufficient to obtain similar ERβ expression levels among all cell lines (Figure 3A) and to mimic constitutive receptor expression, as in our previous study [15]. Extracted RNAs were used for sncRNA sequencing as described in the Materials and Methods section. Analysis of the number of expressed molecules belonging to different sncRNA classes in ERβ+ cell lines of different histotypes demonstrated that the number of miRNAs present in ERβ+ cells reflects that described above for ERβ−, in this case also being lower in MDA-MB-468 cells with respect to the other two cell lines (Supplementary Figure S3A). Concerning other sncRNA subtypes, we observed that the number of detected tRNAs was slightly increased in all cell lines in the presence of ERβ, whereas sncRNAs of all other classes (snoRNA, snRNA, and piRNA) remained invariable (Supplementary Figure S3A). Analysis of highly expressed miRNAs in ERβ+ cells demonstrated that, like in ERβ− cells, the majority (79 molecules) were common between all three cell lines, with the remaining miRNAs common between two cell lines or expressed in a histotype-specific manner (Supplementary Figure S3B). Comparison of the top expressed tRNAs, snoRNAs, snRNAs, and piRNAs among different ERβ-expressing cell lines demonstrated that the number of commonly expressed sncRNAs was similar to that observed in ERβ− cells, comprising 45, 10, and 10 sncRNAs, respectively (Supplementary Figure S3C,D,F), whereas the common snRNA number was significantly lower (4 in ERβ+ vs 14 in ERβ− cells, Supplementary Figure S2C and Figure S3E, respectively). Finally, for each cell line, ERβ-specific changes were determined. A total of 300 sncRNAs (183 up- and 117 down-regulated) were deregulated in basal-like 1 cell line MDA-MB-468, 135 (75 up- and 60 down-regulated) in basal-like 2 cell line HCC1806, and 271 (141 up- and 130 down-regulated) in Hs 578T cells belonging to the claudin-low TNBC subtype (|FC| ≥ 1.5, *p* < 0.05), as shown in Figure 3B,C and Supplementary Figure S4 for miRNAs and all other sncRNAs classes, respectively. Deregulated sncRNAs were mainly subtype-specific, with a low number being common for all three cell line molecules (19 up- and 1 down-regulated sncRNAs) (Figure 3B). In order to understand which molecular processes are regulated by differentially expressed miRNAs, we performed target prediction using miRWalk software [38], focusing on experimentally validated and predicted miRNA-targeted mRNAs, expressed in the presence of ERβ according to our previously published results [15]. Also here, deregulated signaling pathways were mainly cell type-specific, reflecting histotype-specific mechanisms of ERβ action in TNBC (Supplementary Figure S5). In agreement with our previously published results [15], the only group of signaling pathways commonly influenced in all tested cell lines was the cholesterol biosynthesis superpathway, together with all three branches of cholesterol biosynthesis. Interestingly, the IPA functional annotation analysis performed on a set of ERβ-modulated genes, representing miRNA targets, revealed significant similarities between miRNA-targeted functions such as regulation of DNA replication, recombination and repair, gene expression, post-translational modifications, lipid and carbohydrate metabolism, small molecule biochemistry, cellular function, maintenance, morphology, development, movement, assembly and organization and, finally, cell cycle (Figure 3D). All these results confirm our hypothesis that ERβ may exert its oncosuppressive role also through regulation of miRNA expression in TNBC cells.

### 3.4. Upregulation of miR-181a-5p as an Auxiliary Mechanism of ERβ-Induced Cholesterol Biosynthesis Inhibition

In order to evaluate if the ERβ-induced changes of sncRNA profiles observed in TNBC cell lines were also present in TNBC tumors, we performed sncRNA sequencing of RNA samples isolated from 12 ERβ+ and 32 ERβ− TNBC tissues. The samples used for this experiment were characterized by the clinico-pathological features summarized in Table 1. Briefly, the age of TNBC patients ranged between 27 and 77 years for ERβ+ samples and between 24 and 91 years for ERβ− samples. All ERβ+ tissues and 88% (28 samples) of ERβ− ones were at stage III or IV according to the FIGO (International Federation of Gynecology and Obstetrics) staging system, whereas the remaining 12% (4 samples) of ERβ− tissues were classified as stage I or II. Ten (83%) and 23 (72%) ERβ+ and ERβ− TNBC samples were respectively represented by ductal carcinomas, whereas the remainder (2 ERβ+ and 9 ERβ− tissues) were classified as non-ductal ones. Seven (58%) and 5 (42%) ERβ+ patients were characterized by tumor grade 1 and 2 respectively, whereas the grades of 15 (47%), 13 (41%), and 3 (9%) ERβ− samples were 1, 2, and 3 respectively. For one ERβ− sample this information was not available. The development of recurrence was found in 58% and 16% of cases of ERβ+ and ERβ− tissues respectively, whereas this information was not available for 2 ERβ+ and 11 ERβ− patients. Lymph node metastases were found in 42% (5 out of 12) and 47% (15 out of 32) of ERβ+ and ERβ− patients, respectively, and absent in 58% and 53% of ERβ+ and ERβ− patients, respectively. Finally, Ki67 proliferation factor expression was high (>20%) in all ERβ+ patients and 24 (75%) ERβ− ones, and low (<20%) in seven patients whose tumor did not express the receptor. This information was not determined for 1 ERβ−.

**Figure 4:**
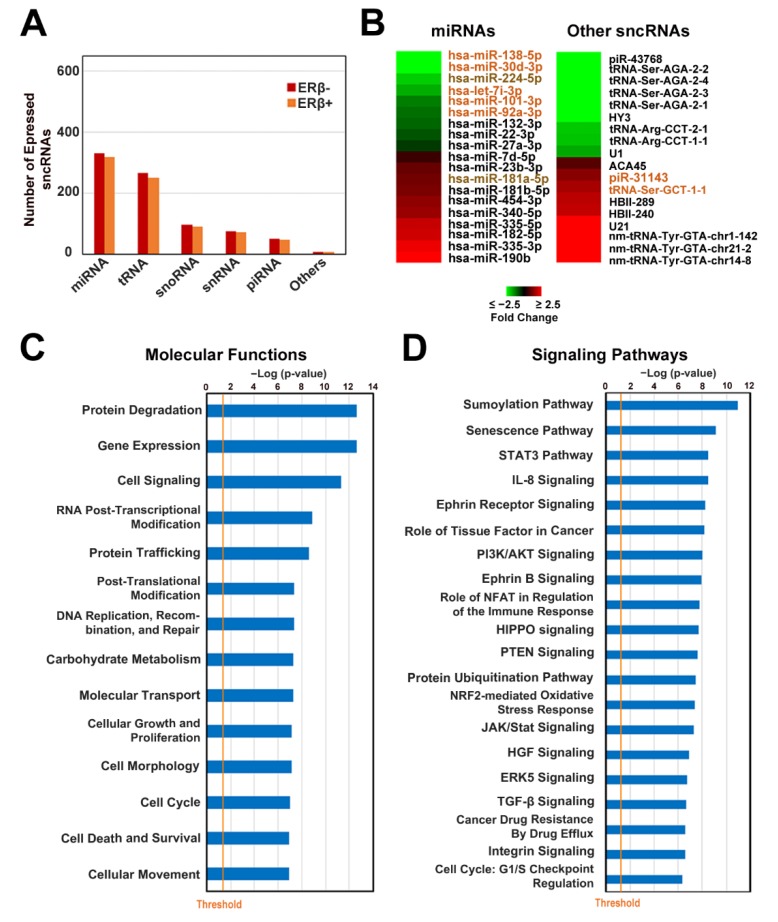
The ERβ-induced change in the sncRNA profile in TNBC tissues and its putative effect on molecular processes. (**A**) Histogram showing the number of miRNAs, tRNAs, snoRNAs, snRNAs, piRNAs, and other sncRNAs detected in ERβ− and ERβ+ tissue samples. Only sncRNAs whose median expression level exceeded a minimum threshold (three normalized reads) are reported. (**B**) Heatmaps showing miRNAs (left) and other sncRNAs (right) deregulated in ERβ+ TNBC tissues (|FC| ≥ 1.3, *p* < 0.05). RNAs differentially expressed in ERβ+ tumor biopsies and three or two cell lines are indicated in brown or orange, respectively. Graphic representation of statistically significant biological functions (**C**) and the top 20 statistically significant signaling pathways (**D**), identified by IPA performed on genes expressed in TNBC tissues and predicted to be targets of miRNAs commonly deregulated both in tissues and at least two of the studied cell lines.

In order to characterize the sncRNA profiles of TNBC tissues, we analyzed the number of expressed sncRNAs of different classes in both ERβ+ and ERβ− samples. We found that the number of identified miRNAs, tRNAs, and snRNAs was slightly lower in tissue samples with respect to cell lines (Figure 4A), whereas snoRNA and piRNA numbers were similar in both types of samples. The number of identified molecules of all sncRNA classes in ERβ+ tissues was slightly lower than in ERβ− ones (Figure 4A). Differential expression analysis revealed deregulation of 37 sncRNAs (|FC| > 1.3, *p* < 0.05) in ERβ+ tissues, among which 19 miRNAs, 10 tRNAs, 4 snoRNAs, 2 snRNAs, and 2 piRNAs were deregulated (Figure 4B). Comparison of ERβ-regulated sncRNAs from tissues with the ones determined using cell culture models revealed 5 miRNAs, 1 tRNA, and 2 piRNAs (Figure 4B) displaying the same behavior in the two cell lines and tissue samples, whereas 2 of the miRNAs—down-regulated miR-224-5p and up-regulated miR-181a-5p (Figure 4B)—were commonly deregulated in all three cell lines and tissue samples, indicating cell-autonomous regulation of their expression by ERβ. Next, in order to evaluate which processes are regulated by the seven identified miRNAs in TNBC tissues, we performed IPA analysis on experimentally validated and predicted miRNA-targeted mRNAs from the miRWalk database [36], considering only genes expressed in TNBC tissues from TCGA database [25]. We found out that the targeted genes are mainly involved in functional processes previously seen to be influenced by ERβ [15]. Indeed, such cell behavior-associated processes as cell growth, proliferation, movement, death, survival, morphology, and cell cycle were found among them (Figure 4C) together with gene expression, RNA post-transcriptional modification, protein degradation and trafficking, post-translational modification, and several other functions, indicating the putative miRNA-mediated regulation of these processes by ERβ. Among the top 20 signaling pathways characterized by the highest statistical significance, many cancer-related pathways were present, for example: sumoylation, STAT3 (Signal transducer and activator of transcription 3), PI3K/AKT (Phosphoinositide 3-kinase/protein kinase B), HIPPO, PTEN (Phosphatase and tensin homolog), TGF-β (Transforming growth factor beta), integrin signaling, and cell cycle regulation via the G_1_/S checkpoint (Figure 4D). In order to evaluate the possible effects of commonly deregulated miRNAs in TNBC cell lines, we referred to a previously published RNA-seq dataset [15] and performed IPA functional analysis on a set of top expressed (values above the third quartile) genes, predicted to be targets of commonly deregulated miRNAs. The results, reported in Supplementary Figure S6, corroborated the data obtained for TNBC tissues and confirmed that multiple signaling pathways were influenced both in cell lines and tissues. Indeed, a comparison of the top 20 highly significant processes found to be influenced in tissues (Figure 4D) and cell lines (Supplementary Figure S6) revealed that eight processes (the sumoylation, senecscence, STAT3, IL-8 (Interleukin 8), HIPPO, PTEN and two ephrin receptor-related signaling pathways) were commonly affected, indicating a high correlation of the results.

**Figure 5:**
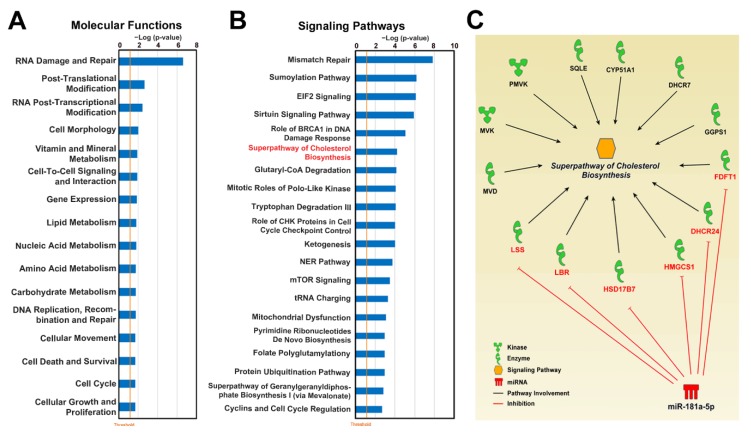
Putative effect of miR-181a-5p deregulation on molecular processes in TNBC tissues. Graphic representation of statistically significant biological functions (**A**) and the top 20 statistically significant signaling pathways (**B**), as identified by IPA performed on a group of genes expressed in TNBC tissues and predicted to be targets of miR-181a-5p. (**C**) Schematic representation of genes participating in cholesterol biosynthesis that were previously found down-regulated by ERβ in TNBC cells [15], with miR-181a-5p targets shown in red.

To understand which of the deregulated miRNAs in both TNBC cell lines and tissues most likely assist ERβ in exerting its oncosupressive effects, we first evaluated the expression level of all seven miRNAs and found out that four of them, down-regulated miR-138-5p, miR-30d-3p, miR-224-5p, and miR-7i-3p, were expressed at a low level in TNBC cells and were not present among the top expressed miRNAs (Supplementary Figure S1), indicating the minor impact of their deregulation on TNBC transcriptome. The remaining three miRNAs—miR-101-3p, miR-92a-3p and miR-181a-5p—instead occupied the 8th, 11th, and 51st positions, respectively (Supplementary Figure S1), suggesting the high possibility that their deregulation may cause a profound effect on molecular processes in TNBC cells. Further, as the expression level of miR-92a-3p and miR-181a-5p was at least eight times higher with respect to that of miR-101-3p, we decided to focus our attention on these two miRNAs in our further research. Surprisingly, we found out that miR-92a-3p is known to regulate ERβ expression in BC [70], whereas its role in TNBC has not been investigated yet. The effect of miR-181a-5p in TNBC instead is ambiguous, due to the fact that controversial results are available in the literature [71]. In order to reveal whether one of these two miRNAs may regulate molecular processes known to be deregulated in TNBC by ERβ (e.g., cholesterol biosynthesis), we performed IPA functional and signaling pathway analysis on a set of genes representing putative miRNA targets expressed in TNBC tissues. We found that both miRNAs regulate multiple molecular functions involved in cell behavior such as cellular growth, proliferation, movement, death and survival, post-translational modification, RNA post-transcriptional modification, and gene expression (Figure 5A, Supplementary Figure S7). Analysis of influenced signaling pathways revealed that in case of miR-181a-5p, superpathway of cholesterol biosynthesis was among the top 10 the most significant signaling pathways (Figure 5B) indicating the possibility of down-regulation of this process by up-regulated miR-181a-5p. In order to verify the finding, we performed the same analysis using a set of putative miRNA-targeted genes deregulated in the presence of ERβ in TNBC cell lines, and found that the only group of commonly deregulated signaling pathways was that of cholesterol biosynthesis, including all four branches (Supplementary Figure S8), pointing to the possible role of miRNA-181a-5p in the regulation of this metabolic process.

In conclusion, according to the obtained results, upregulation of miR-181a-5p potentially leads to inhibition of all branches of the cholesterol biosynthesis pathway by targeting expression of six genes (Figure 5C) and may represent an auxiliary mechanism of ERβ-induced cholesterol biosynthesis regulation, complementary to the one described in our previous study [15].

It should be noted that the use of nominal *p*-values for identification of deregulated miRNAs here represents a limitation, since it could lead to increase of the false positive rate that could have been corrected by increasing the number of measurements performed in cell lines and the tumor samples analyzed. Given the relatively low number of molecules detected by sncRNA sequencing, a rigorous statistical analysis would in this case have caused the loss of relevant information and for this reason, unequivocal identification of the full set of miRNAs controlled by ERβ in TNBC cells will require further studies involving more time points in tumor samples.

## 4. Discussion

Triple-negative breast cancer is an extremely aggressive disease that lacks targets commonly used for breast cancer treatment, and is characterized by a distinct molecular profile [2]. miRNA dysregulation is known as an important mechanism of post-transcriptional regulation of gene expression implicated in TNBC [72]. Here, using sncRNA sequencing, we investigated the small non-coding RNA expression profiles of three TNBC cell lines that belong to different TNBC subtypes. Comparison of the lists of highly expressed miRNAs identified in analyzed cells revealed seven molecules, whose oncogenic properties were already associated with this cancer type, that may represent a novel molecular biomarkers or therapeutic targets for treatment of this disease. Indeed, cell viability and epithelial–mesenchymal transition-promoting properties of miR-221-3p were already described, together with its ability to induce cell proliferation of TNBC cells [73], a characteristic feature known also for another four highly expressed miRNAs: miR-181b-5p, miR-21-3p, miR-25-3p, and miR-27a-3p [44,47,74,75]. Moreover, highly expressed miRNAs miR-103-3p, miR-181b-5p, miR-20a-5p, miR-21a-3p, miR-221-3p, and miR-27a-3p are involved in regulation of TNBC cells migration [44,45,46,73,74,75], whereas miR-103-3p, miR-20a-5p, and miR-27a-3p have been previously associated with invasion of TNBC cells [45,46,75]. A high expression of another two miRNAs, miR-221-3p and miR-27a-3p, was previously correlated with poor TNBC patient survival [40,42]. Moreover, expression of miR-181b-5p represents a common feature of aggressive breast cancers [74], whereas another two miRNAs—miR-103-3p and miR-20a-5p—were found to be highly expressed in both TNBC tissues and cell lines [45,46]. Finally, it was demonstrated that overexpression of miR-27a-3p and miR-181b-5p confers chemoresistance to PARP (poly ADP ribose polymerase) inhibitors [39] and neutralizes the ionizing radiation effect [43,75], respectively. Altogether, these data indicate that the sncRNA expression dataset generated here represents a valuable source of information for further evaluation of potential of aforementioned miRNAs and other molecules as molecular biomarkers of TNBC. The role of ERβ in cancer progression is poorly understood, mainly due to the fact that the major part of commercially available anti-ERβ antibodies are characterized by low specificity, leading to misguiding results available in the literature. Recently, in a study by Andersson et al. the specificity of 13 antibodies was extensively validated and it was demonstrated that only one, PPZ0506, specifically recognizes this receptor and is applicable to immunohistochemistry staining using human cells and tissues [12]. However, using that antibody authors did not detect ERβ expression in breast cancer tissues. Nonetheless, further studies performed using the same antibody on a larger group of breast cancer tissues, including our study, confirmed its expression both in normal and cancerous breast tissues [15,76]. Moreover, using cell lines belonging to three different histotypes, we demonstrated that ERβ exerts an antiproliferative effect on TNBC, reduces the migratory and clonogenic potential of these cells and, finally, inhibits cell cycle progression [15].

Here, in order to expand our previous research and to reveal supplementary molecular mechanisms of ERβ-induced regulation of gene expression, we used three TNBC cell lines where a full-length ERβ cDNA sequence under control of inducible promoter was previously introduced [15]. A comparison of sncRNA profiles of ERβ+ cells and the corresponding ERβ− cells revealed histotype-specific changes of sncRNA expression in the presence of ERβ; a similar effect was observed when analyzing ERβ-induced RNA expression changes in the same cells [15]. Also in this case, analysis of affected signaling processes performed on a lists of deregulated miRNA targets revealed mainly histotype-specific changes, with the only common group of signaling pathways consisting of all branches of cholesterol biosynthesis, in line with our previous results concerning ERβ-induced inhibition of cholesterol biosynthesis in TNBC [15]. Moreover, one recently published study demonstrated that cholesterol biosynthesis is essential for breast cancer stem cell propagation [77], whereas another showed that the hyper-activated cholesterol biosynthesis program of TNBC is regulated by nuclear receptor RORγ (RAR-related orphan receptor gamma), whose inhibition by receptor antagonists leads to tumor regression in patient-derived xenografts and immune-intact models [78], confirming the extreme importance of cholesterol biosynthesis in TNBC cancer progression.

Finally, we performed sncRNA profiling of ERβ+ and ERβ− TNBC tissues, where receptor expression was assayed with a validated PPZ0506 antibody [12]. Comparison of in vitro and in vivo-observed sncRNA expression changes revealed that miR-92a-3p and miR-181a-5p were commonly deregulated in at least two ERβ-expressing cell lines and tissues, representing cell-autonomous changes. Moreover, both of them were present among the top expressed miRNAs, indicating the possibility of significant impact of their deregulation on molecular processes in TNBC. Bibliographic research about the role of miR-92a-3p and miR-181a-5p in BC and particularly in TNBC demonstrated that whereas miR-92a-3p has not been studied yet, in breast cancer it regulates ERβ expression, as a significant negative correlation between its expression and both ERβ mRNA and protein expression levels was demonstrated on a cohort of primary breast tumors [70]. Moreover, miR-92-3p expression targeting, aimed at re-activating expression of ERβ, was suggested as a therapeutic strategy against hormone-responsive breast cancer [70]. In line with these results, we observed that miR-92a-3p was down-regulated in ERβ+ TNBC tissues and basal-like cells (Figure 4B).

Although the role of miR-181a-5p in TNBC has been extensively studied, it is not yet completely clear whether it exerts oncosuppressive or oncogenic functions, as there is evidence supporting both effects. Thus, it was observed that miR-181a-5p expression was down-regulated in TNBC tumor samples compared to corresponding normal breast tissues [79] and was down-regulated together with other seven miRNAs in patients with lymph-node metastasis when compared to the group of patients without them [80]. At the same time, increased expression of miR-181a-5p was found to correlate with TNBC [61,63], and grade 3 tumors showed the highest expression of this miRNA compared to grade 1 and 2 tumors [39]. Next, in TNBC cell line MDA-MB-231 and SK-3rd breast cancer stem cells, the miR-181a-5p expression level was significantly lower than that of the hormone-responsive MCF-7 cell line, characterized by less aggressive behavior [81], whereas Niu et al. reported that miR-181a expression induction promotes chemotherapeutic resistance and metastasis [62]. Here, the application of IPA signaling pathway analysis on a subset of miR-181a-5p-targeted genes revealed that multiple genes participating cholesterol biosynthesis represent putative targets of this miRNA, indicating the possibility of miR-181a-5p-mediated regulation of this metabolic process in TNBC cells. Altogether, these data indicate that further research is needed to clarify the role of miR-181a-5p in TNBC and that ERβ is involved not only in recruitment of multiple chromatin-modifying complexes to novel genome locations but also in miRNA-mediated regulation of gene expression in TNBC.

## 5. Conclusions

In conclusion, the results of this study demonstrate that sncRNAs are key molecules governing TNBC behaviors and that ERβ can influence the final biological outcome of this tumor subtype by modulating post-transcriptional events. These findings provide new leads toward understanding the oncosuppressive role of ERβ via post-transcriptional control of RNA activity. These results will require further validation but suggest that specific sncRNA expression and deregulation represent a molecular signature of potential usefulness in the assessment of progression, follow-up, and prognosis of this disease.

## Figures and Tables

**Table 1:** Clinicopathologic features of the tissue samples from 44 patients affected with TNBC, analyzed by sncRNA sequencing.

Characteristics	ERβ+	ERβ−
**Number**	12 (27%)	32 (73%)
**Age (Years)**		
Median (range)	62 (27–77)	62 (24–91)
**Stage (FIGO)**		
I–II	0	4 (12%)
III–IV	12 (100%)	28 (88%)
**Histotype**		
IDC	9 (75%)	23 (72%)
IDLC	1 (8%)	0
ILC	2 (17%)	3 (9%)
AC	0	2 (6%)
Others	0	4 (13%)
**Histological Grade**		
G1	7 (58%)	15 (47%)
G2	5 (42%)	13 (41%)
G3	0	3 (9%)
NA	0	1 (3%)
**Disease Progression**		
No progression	3 (25%)	16 (50%)
Progression	7 (58%)	5 (16%)
NA	2 (17%)	11 (34%)
**Lymph Node Metastasis**		
Negative	7 (58%)	17 (53%)
Positive	5 (42%)	15 (47%)
**Ki67**		
<20%	0	7 (22%)
>20%	12 (100%)	24 (75%)
ND	0	1 (3%)

FIGO—International Federation of Gynecology and Obstetrics, IDC—invasive ductal carcinoma, ILC—invasive lobular carcinoma, IDLC—mixed invasive ductal/lobular carcinoma, AC—atypic carcinoma, Others—other carcinoma types, ND—not determined, NA—not available.

## Supplementary Materials

The following are available online at https://www.mdpi.com/2073-4409/9/4/874/s1, Figure S1: Comparison of the top expressed miRNAs in three TNBC cell lines; Figure S2: Comparison of sncRNA expression profiles in the three TNBC cell lines studied; Figure S3: Characterization of sncRNAs expression profiles in ERβ−expressing TNBC cell lines; Figure S4: Effects of ERβ on sncRNA expression profiles in TNBC cell lines; Figure S5: Analysis of signaling pathways downstream of miRNAs differentially expressed in response to ERβ in TNBC cells; Figure S6: Putative effect of the deregulation of seven mRNAs on molecular processes in TNBC cell lines; Figure S7: Putative effect of miR-92a-3p deregulation on molecular processes in TNBC tissues; Figure S8: Functional analysis of the downstream effects of miR-181a-5p in TNBC cell lines; Table S1: sncRNAs differentially expressed in ERβ+ vs ERβ− MDA-MB-468 cells; Table S2: sncRNAs differentially expressed in ERβ+ vs ERβ− HCC1806 cells; Table S3: sncRNAs differentially expressed in ERβ+ vs ERβ− Hs 578T cells; Table S4: sncRNAs differentially expressed in ERβ+ vs ERβ− TNBC biopsies.
