# Supplementary material for: Small Non-Coding RNA Profiling Identifies miR-181a-5p as a Mediator of Estrogen Receptor Beta-Induced Inhibition of Cholesterol Biosynthesis in Triple-Negative Breast Cancer
Source: Cells. 2020 Apr 3;9(4):874. doi: 10.3390/cells9040874 (PMC7226848; doi:10.3390/cells9040874)
Supplement: Supplementary file 1 [file cells-09-00874-s001.zip › Alexandrova_et_al_Cells_Supplementary_Files/Alexandrova_et_al_Cells_Supplementary_Materials_2Mar20.pdf]

SUPPLEMENTARY MATERIALS

Small non-coding RNA profiling identifies miR-181a as a mediator of Estrogen Receptor beta-induced inhibition of cholesterol biosynthesis in triple-negative breast cancer

Elena Alexandrova, Jessica Lamberti, Pasquale Saggese, Giovanni Pecoraro, Domenico Memoli, Valeria Mirici Cappa, Maria Ravo, Roberta Iorio, Roberta Tarallo, Francesca Rizzo, Francesca Collina, Monica Cantile, Maurizio Di Bonito, Gerardo Botti, Giovanni Nassa, Alessandro Weisz and Giorgio Giurato

Supplementary Figures S1-S7

FIGURE LEGENDS

Supplementary Figure S1

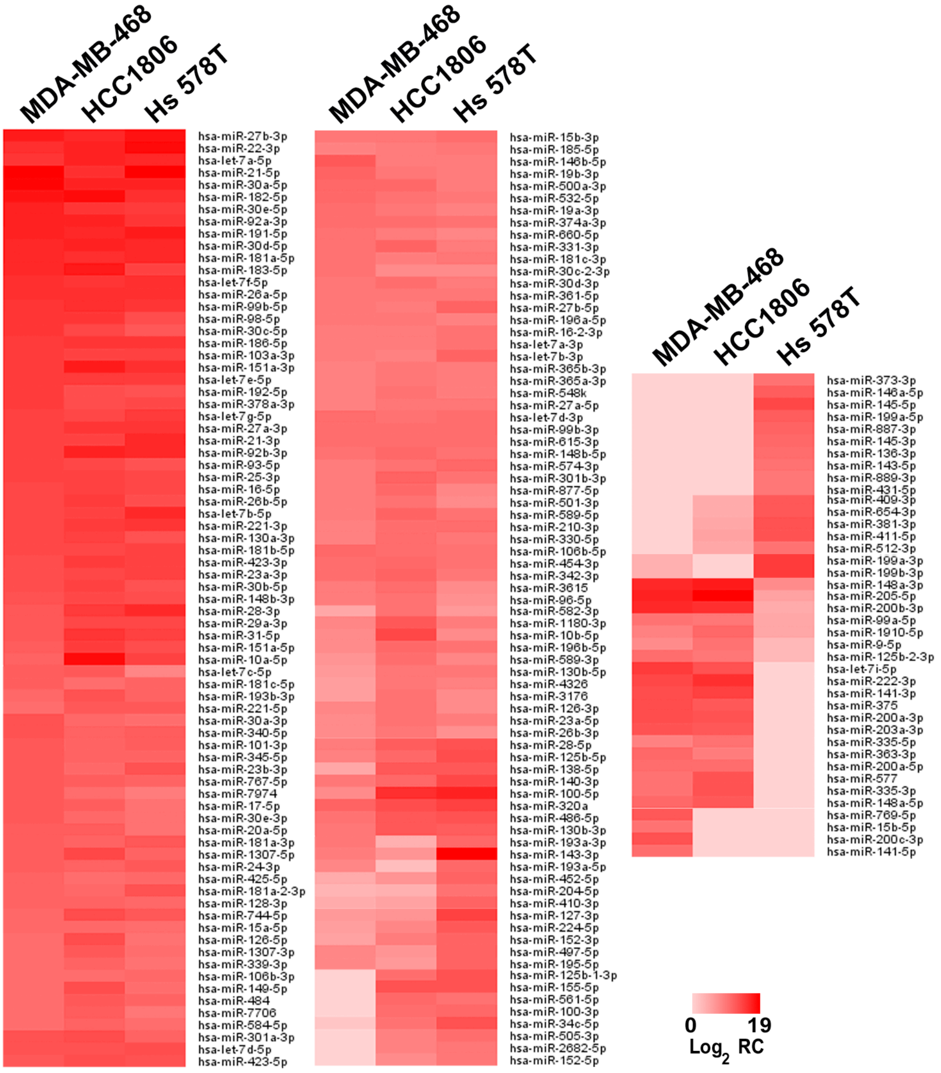

Supplementary Figure S1. Comparison of the top expressed miRNAs in three TNBC cell lines. Heatmap showing expression intensity (Log_2 of Normalized Read Count) of 194 miRNAs, whose expression level was above the third quartile in at least one of the TNBC cell lines analyzed.

Supplementary Figure S2

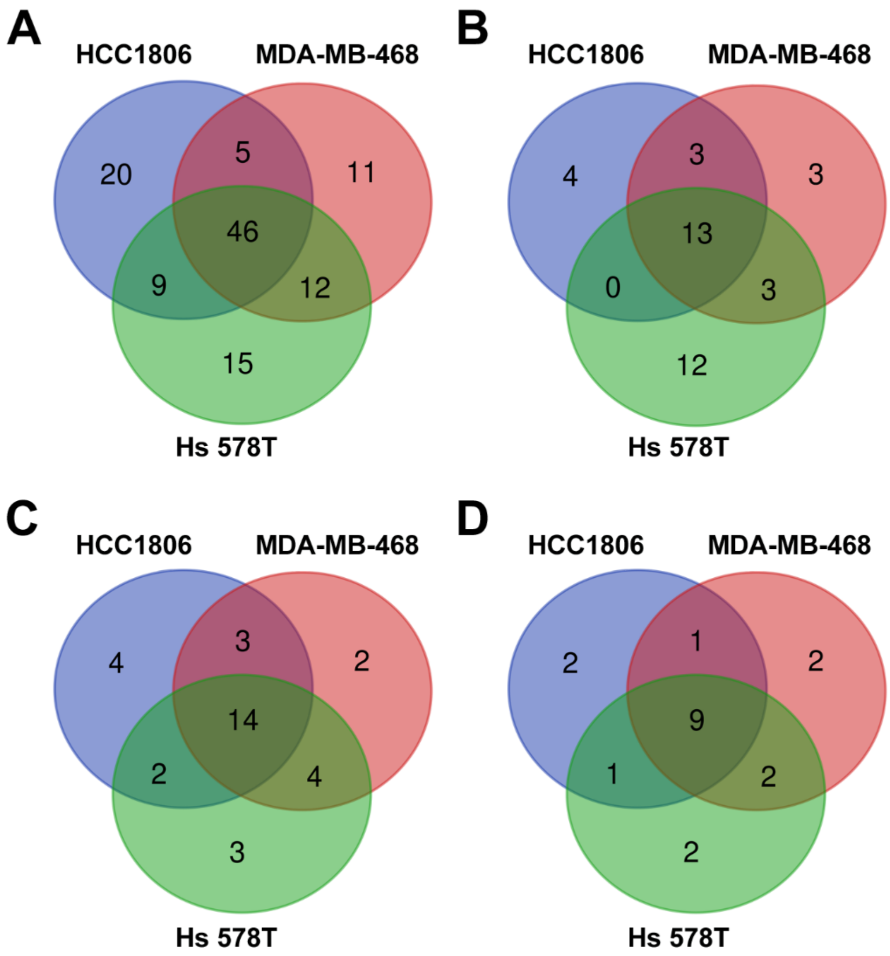

Supplementary Figure S2. Comparison of sncRNA expression changes in response to ERβ in the three TNBC cell lines studied. Venn diagram showing the number of common and specific tRNAs (**A**), snoRNAs (**B**), snRNAs (**C**) and piRNAs (**D**), highly expressed in TNBC cell lines (whose expression level was above the third quartile of normalized read count in considered sncRNA group).

Supplementary Figure S3

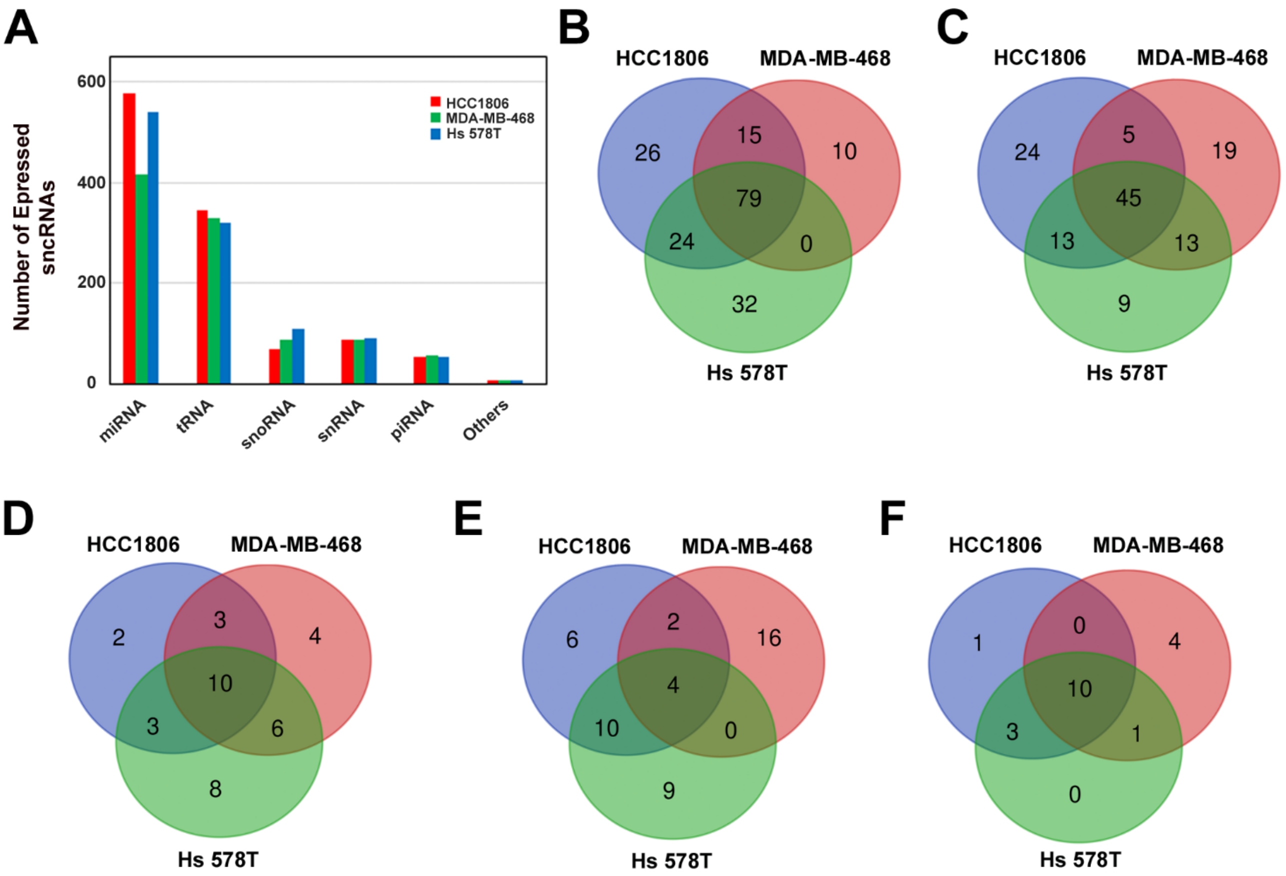

Supplementary Figure S3. Characterization of sncRNAs expression changes in response to ERβ in TNBC cell lines. (**A**) Histogram showing the number of expressed miRNAs, tRNAs, snoRNAs, snRNAs, piRNAs and other sncRNAs whose expression level exceeded 3 normalized read count is reported. Venn diagrams showing the number of common and specific miRNAs (**B**), tRNAs (**C**), snoRNAs (**D**), snRNAs (**E**) and piRNAs (**F**), highly expressed in TNBC cell lines (whose expression level was above the third quartile in considered sncRNA group).

Supplementary Figure S4

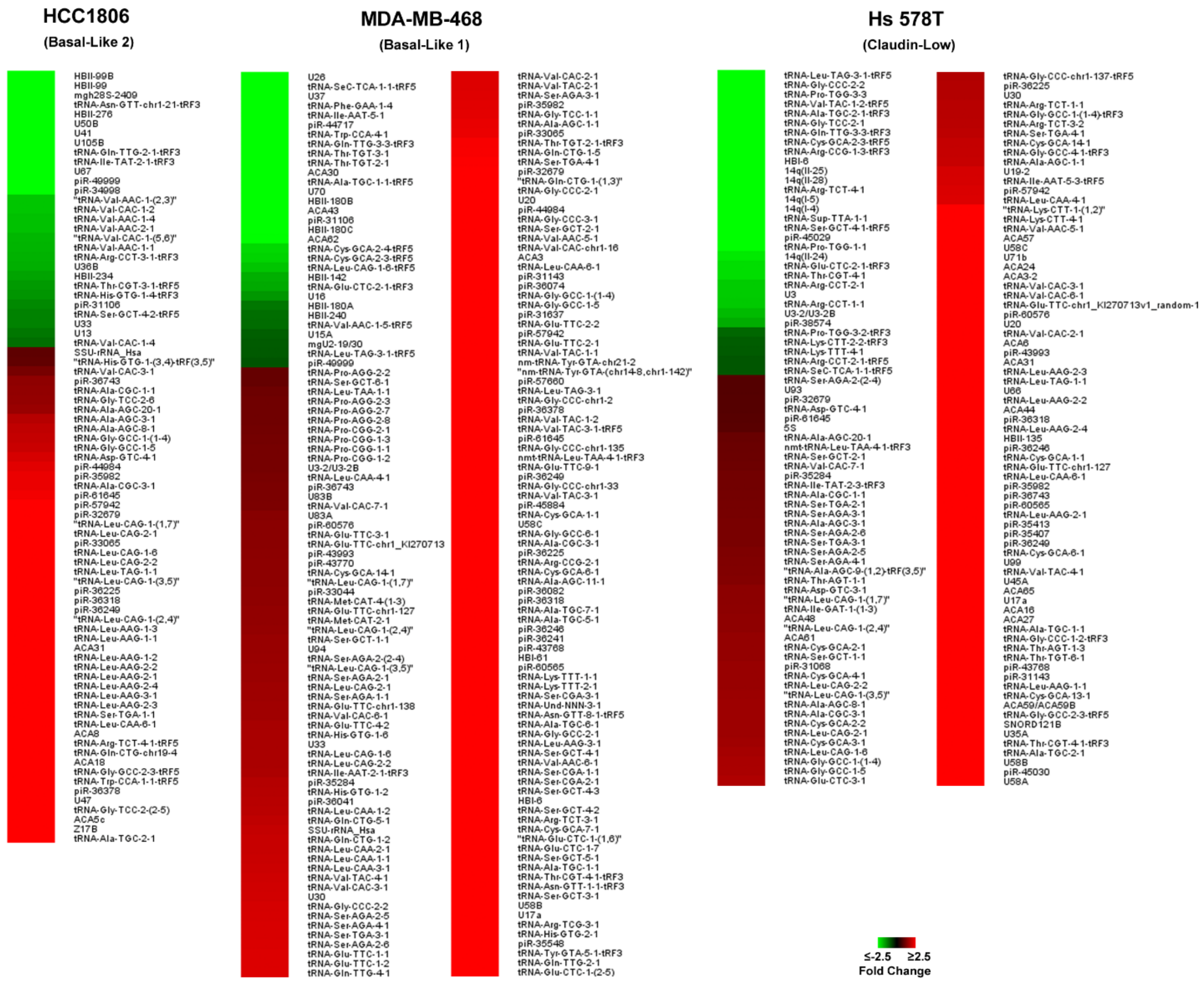

Supplementary Figure S4. Effects of ERβ on sncRNA expression profiles in TNBC cell lines. The heatmaps showing ERβ-regulated sncRNAs in indicated cell lines ($|FC| \geq 1.5$, p-value < 0.05)

Supplementary Figure S5

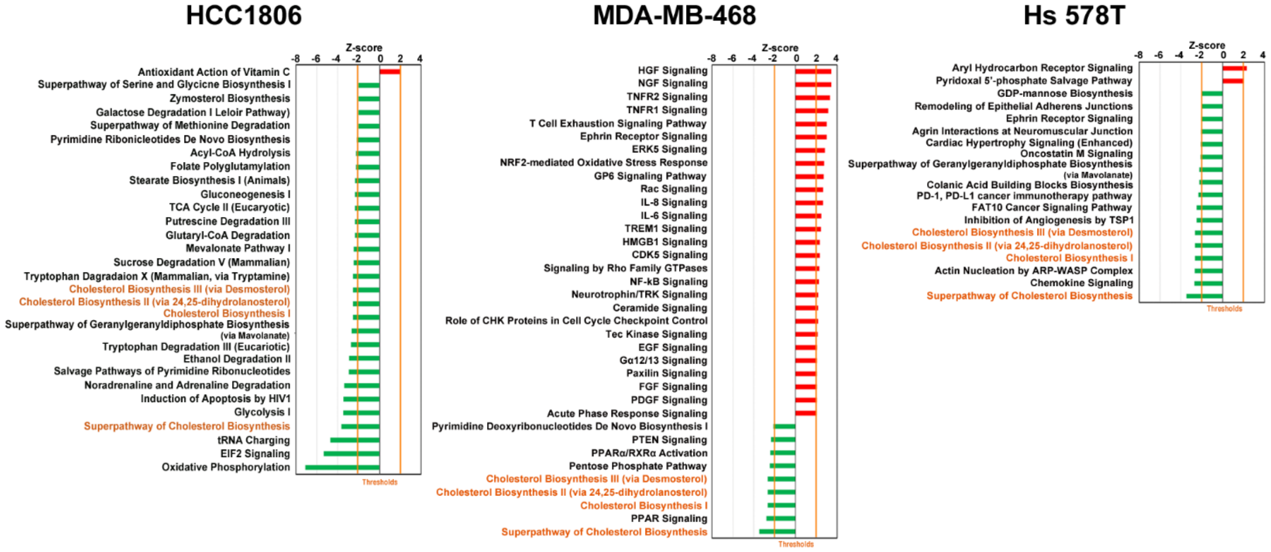

Supplementary Figure S5. Analysis of signaling pathways downstream of miRNAs differentially expressed in response to ERβ in TNBC cells. IPA signaling pathway analysis performed on ERβ-modulated genes, predicted to be targets of differentially expressed miRNAs ($|FC| \geq 1.5$, p-value < 0.05) in corresponding cell lines. Names of commonly deregulated in all three cell lines signaling pathways are indicated in orange. The vertical orange line indicates utilized Z-score threshold ($|Z\text{-score}| \geq 2$).

Supplementary Figure S6

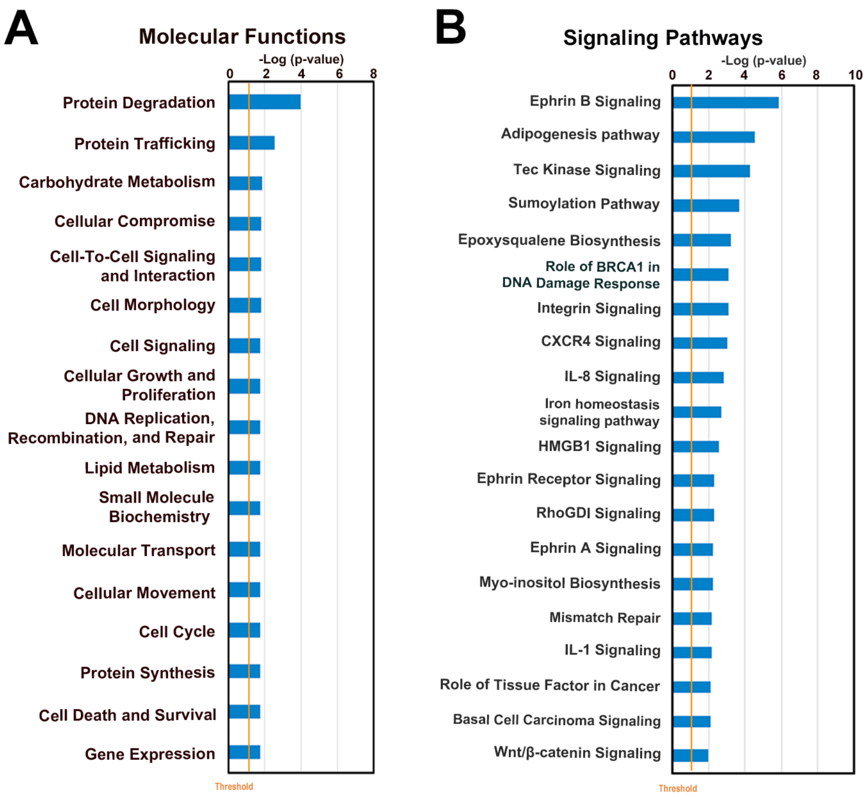

Supplementary Figure S6. Putative effect of miR-92a-3p deregulation on molecular processes in TNBC tissues. Graphic representation of statistically significant biological functions (A) and top 20 statistically significant signaling pathways (B), identified by IPA, performed on a group of genes, expressed in TNBC tissues and predicted to be targets of miR-181a-5p.

Supplementary Figure S7

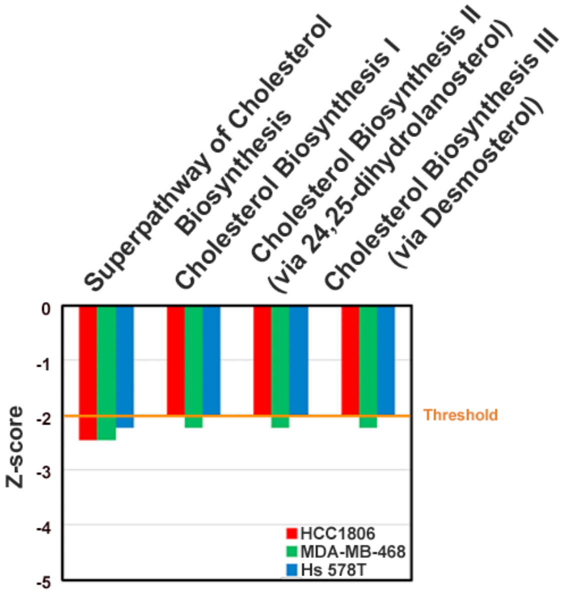

Supplementary Figure S7. Functional analysis of the downstream effects of miR-181a in TNBC cell lines. Graphic representation of statistically significant pathways, identified by IPA, performed on a group of ERβ-regulated genes, predicted to be targeted by miR-181a-5p. Only signaling pathways, commonly deregulated in all three cell lines, are shown. The horizontal orange lines indicate Z-score threshold (|Z-score| ≥ 2).
